# A Proteomics Profiling Reveals the Neuroprotective Effects of Melatonin on Exogenous β‐amyloid‐42 Induced Mitochondrial Impairment, Intracellular β‐amyloid Accumulation and Tau Hyperphosphorylation in Human SH‐SY5Y Cells

**DOI:** 10.1002/cbin.70013

**Published:** 2025-03-06

**Authors:** Jiraporn Panmanee, Matthew Phanchana, Phorutai Pearngam, Nopphon Petchyam, Kornkanok Promthep, Ponlawit Wisomka, Suchanoot Kutpruek, Supitcha Pannengpetch, Tanya Prasertporn, Sujira Mukda, Piyarat Govitrapong, Chutikorn Nopparat

**Affiliations:** ^1^ Research Center for Neuroscience, Institute of Molecular Biosciences Mahidol University Nakhon Pathom Thailand; ^2^ Department of Molecular Tropical Medicine and Genetics, Faculty of Tropical Medicine Mahidol University Bangkok Thailand; ^3^ Biological Sciences Program Mahidol University International College, Mahidol University Nakhon Pathom Thailand; ^4^ Center for Advanced Therapeutics, Institute of Molecular Biosciences Mahidol University Nakhon Pathom Thailand; ^5^ Chulabhorn Graduate Institute, Chulabhorn Royal Academy, Laksi Bangkok Thailand; ^6^ Center for Research and Innovation, Faculty of Medical Technology Mahidol University Nakhon Pathom Thailand; ^7^ Innovative Learning Center Srinakharinwirot University Bangkok Thailand

**Keywords:** Alzheimer's disease, beta‐amyloid, melatonin, mitochondria, neurofibrillary tangle

## Abstract

Alzheimer's disease (AD) is prevalent in the elderly population and characterized by the intracellular accumulation of neurofibrillary tangles (NFTs), composed of tau proteins, and extracellular deposition of beta‐amyloid protein (Aβ). The present study aimed to investigate the neuroprotective effects of melatonin on Aβ42‐induced AD‐like pathology in SH‐SY5Y cell lines. To assess the effects of melatonin on Aβ42‐exposed cells, we performed a proteomics analysis of altered protein expression in Aβ42‐treated cells, with or without melatonin Pretreatment, using label‐free nano‐LC‐MS/MS. Experimental validations of pathways related to the neuroprotective effects of melatonin were carried out using Milliplex amyloid beta and tau magnetic bead assays, Western blot analysis, and measurements of mitochondrial membrane potential and ROS levels. Our results show that Aβ42 exposure led to an increase in an accumulation of intracellular Aβ42/40 and phosphorylated tau (Thr181)/Tau ratios. Pretreatment with melatonin effectively reduced the levels of these pathogenic proteins. Proteomics analysis has revealed protein markers associated with the Alzheimer's disease pathway, neuronal synapses, cellular apoptosis, and mitochondrial functions. Changes in proteins regulating the mitochondrial permeability transition pore, the electron transport chain, and mitochondrial oxidative stress were observed in Aβ42‐treated cells. Pretreatment with melatonin protected the cells against Aβ42‐induced cellular damages by regulating the expression of several proteins underpinning these biological processes, including the suppression of mitochondrial ROS generation and mitigation of mitochondrial membrane depolarization.

**Abbreviations**
AD: Alzheimer's disease
AIFM3: Apoptosis‐inducing factor, mitochondrion‐associated 3
APP: Amyloid precursor protein
ARHGEF7: Rho guanine nucleotide exchange factor 7
ATP5B: ATP synthase subunit beta
Aβ: Beta‐amyloid
BBC3: BCL2 binding component 3
CANX: Calnexin
CEP131: Centrosomal protein of 131 kD
CKAP5: Cytoskeleton associated protein 5
CLASP1: Cytoplasmic linker associated protein 1
COX8C: Cytochrome c oxidase subunit 8 C
CSF: cerebrospinal fluid
DDR1: Epithelial discoidin domain‐containing receptor 1
DNAH8: Dynein heavy chain 8
FDX1: Ferredoxin 1
FRS3: Fibroblast growth factor receptor substrate 3
GLRX5: Glutaredoxin 5
GRIN1: N‐methyl‐d‐aspartate (NMDA) receptor subunit 1
GRIN2B: N‐methyl‐d‐aspartate (NMDA) receptor subunit 2B
GRIN2D: N‐methyl‐d‐aspartate (NMDA) receptor subunit 2D
KIF19: Kinesin‐like protein 19
MAP2K2: Mitogen‐activated protein kinase kinase 2 or MEK2
MAP6D1: MAP6 domain containing 1
MARC2: MARC2‐mitochondrial amidoxime reducing component 2
MCUR1: Mitochondrial calcium uniporter regulator 1
MERTK: Tyrosine‐protein kinase Mer
MPG: N‐methylpurine DNA glycosylase
mPTP: mitochondrial permeability transition pore
MRPL52: Mitochondrial ribosomal protein L52
MT: Microtubule
NFTs: Neurofibrillary tangles
NTNG2: Netrin‐G2
PARP: cleaved‐poly (ADP‐ribose) polymerase
PLCB3: Phospholipase C beta 3
PPIF: Peptidyl‐prolyl cis–trans isomerase F, mitochondrial
PRDX3: Peroxiredoxin 3
RHOT1: Ras homolog family member T1
ROS: Reactive oxygen species
SCO1: Protein SCO1 homolog, mitochondrial.
SCO2: Synthesis of Cytochrome c Oxidase 2
SHC1: SHC‐transforming protein 1

## 1 Introduction

Melatonin, a neurohormone produced by the pineal gland, regulates sleep‐wake cycles (Skene 2003). The antioxidant and anti‐inflammatory properties of melatonin have been extensively studied (Reiter et al. 2010; Tarocco et al. 2019). As an antioxidant, melatonin can neutralize free radicals and reactive oxygen species (ROS) that cause oxidative damage to cells and tissues (Reiter et al. 2016). Melatonin also stimulates the activities of antioxidant enzymes, such as superoxide dismutase and glutathione peroxidase, which help to protect cells from oxidative stress (Reiter et al. 2016, 2010). Owing to its versatile properties, melatonin is considered a promising therapeutic agent for a wide range of conditions associated with oxidative stress and inflammation, such as neurodegenerative diseases, cardiovascular diseases, and cancer (Chen et al. 2020; Wongprayoon and Govitrapong 2020). There is growing evidence suggesting that melatonin levels in the blood may be lower in individuals with Alzheimer's disease (AD) compared to healthy individuals (Cardinali et al. 2014).

AD is a progressive neurodegenerative disease and the most prevalent cause of dementia in elderly individuals. Patients with AD experience memory loss and cognitive deterioration, which worsen as the disease advances (DeTure and Dickson 2019). AD is characterized by the presence of extracellular beta‐amyloid (Aβ) plaques and intracellular neurofibrillary tangles (NFTs), both of which are caused by the accumulation of misfolded Aβ peptides and abnormally hyperphosphorylated tau, respectively (Castellani et al. 2010). Aβ is a product of amyloid precursor protein (APP) metabolism, whose function is elusive but presumed to be important in neuronal growth, synaptogenesis, and synaptic function that correlate with learning and memory (Müller et al. 2017). APP is sequentially cleaved first by β‐secretases and then by γ‐secretases, yielding Aβ peptides (O'Brien and Wong 2011). Aβ40 and Aβ42 are the two most common isoforms of APP cleavage products, of which Aβ42 is predisposed to form oligomers, fibrils, and plaques in AD brains (Viola and Klein 2015). In addition, the levels of soluble Aβ42 increase in the brain, but decrease in cerebrospinal fluid (CSF), hence Aβ42 being considered an AD biomarker (Nirmalraj et al. 2023). Aβ42 aggregates more rapidly than Aβ40 and was found to be the predominant isoform deposited in diffuse plaques, which suggests a crucial role in the early stages of AD pathology (Murphy and LeVine 2010). The elevation in the Aβ42/40 ratio in the brain reflects the deposition of Aβ42 into amyloid plaques and the altered processing of Aβ peptides (Hampel et al. 2021). Conversely, tau proteins belong to a microtubule (MT)‐associated proteins class primarily found in neurons, where they facilitate the assembly of tubulin monomers into microtubules which constitute the neuronal MT network. This network is crucial in maintaining cell morphology and providing a scaffold for axonal transport (Buée et al. 2000). A loss of tau function may result in a disturbance of the MT network, a characteristic observed in a class of related disorders known as tauopathies such as AD (Barbier et al. 2019). The presence of hyperphosphorylated tau (pTau) in the CSF is linked to NFTs in AD (Schraen‐Maschke et al. 2008), and higher levels of total tau are linked to neurodegeneration (Rawat et al. 2022; Sergeant et al. 2008). pTau (Thr181) is widely used as a biomarker for diagnosing and monitoring AD progression, and links to diffuse cortical Aβ pathology (Gonzalez‐Ortiz et al. 2023; Lussier et al. 2021).

Mitochondrial dysfunction tends to play a significant role in the early‐onset and progression of AD (Perez Ortiz and Swerdlow 2019). Mitochondrial dysfunction in AD may be related to impaired mitochondrial biogenesis, mitophagy, and changes in mitochondrial dynamics (Swerdlow 2018; Wang et al. 2014). These changes can lead to the accumulation of dysfunctional mitochondria and oxidative stress, which can further damage neurons and contribute to AD progression (Wang et al. 2014). Multiple studies have shown that melatonin treatment improves mitochondrial function and reduces oxidative stress in individuals with AD‐related mitochondrial dysfunction (Dragicevic et al. 2011). Previous studies demonstrated that melatonin treatment was able to both restore mitochondrial function and reduce oxidative stress, while also improving cognitive performance (Cardinali et al. 2014; Roy et al. 2022).

Recently, the presence of intraneuronal Aβ accumulation in the brain has been described, which suggests a possible association between intraneuronal Aβ accumulation and the development of synaptic pathology and plaques in AD (Gouras et al. 2010). Moreover, this intracellular Aβ accumulation has been shown to increase levels of tau hyperphosphorylation, and trigger synaptic and mitochondrial dysfunction (Pavlov et al. 2009). This study aims to investigate the potential neuroprotective effects of melatonin on exogenous Aβ42 exposure in human neuroblastoma SH‐SY5Y cell lines. We hypothesized that Aβ42 exposure may exacerbate intracellular Aβ accumulation and tau protein phosphorylation. Additionally, a proteomics analysis was conducted to evaluate the impact of melatonin on protein levels related to AD pathways, synapses, and mitochondrial functions potentially caused by these accumulations. These findings may contribute to the development of melatonin‐based therapeutics for the prevention and treatment of AD.

## 2 Materials and Methods

### 2.1 Chemicals

Minimum essential medium (MEM), Ham's F‐12 medium, fetal bovine serum (FBS), penicillin, and streptomycin were purchased from Gibco BRL (Gaithersburg, MD, USA). Melatonin was purchased from Sigma‐Aldrich (St Louis, MO, USA); Aβ42 from Anaspec (Fremont, CA, USA); antibodies, including cleaved PARP (Asp214) rabbit mAb (#5625), cleaved caspase‐3 (Asp175) rabbit mAb (#9661), vinculin rabbit mAb (#13901), NMDA receptor 2 A (GluN2A) rabbit mAb (#4205), phospho‐GluN2A (Tyr1246) rabbit mAb (#4206), GluN2B rabbit mAb (#4212), and phospho‐GluN2B (Tyr1472) rabbit mAb (#4208) from Cell Signaling Technology (Danvers, MA, USA); Clarity™ Western ECL substrate (#1705060) from Bio‐Rad Laboratories (Hercules, CA, USA); MitoSOX™ mitochondrial superoxide indicators (#M36008) from Invitrogen™, Thermo Fisher Scientific (Waltham, MA USA); JC‐1 – Mitochondrial Membrane Potential Assay Kit (#ab113850) from Abcam (Cambridge, UK); MILLIPLEX Human Amyloid Beta and Tau Magnetic Bead Panel (#HNABTMAG‐68K) from Millipore, Merck (Darmstadt, Germany); phosphate buffered saline (PBS) from Sartorius (Goettingen, Germany); Muse^TM^ Annexin‐V & Dead Cell Assay Kit from Luminex (Austin, TX, USA).

### 2.2 Cell Culture and Aβ42 Treatment

**Figure 1:**
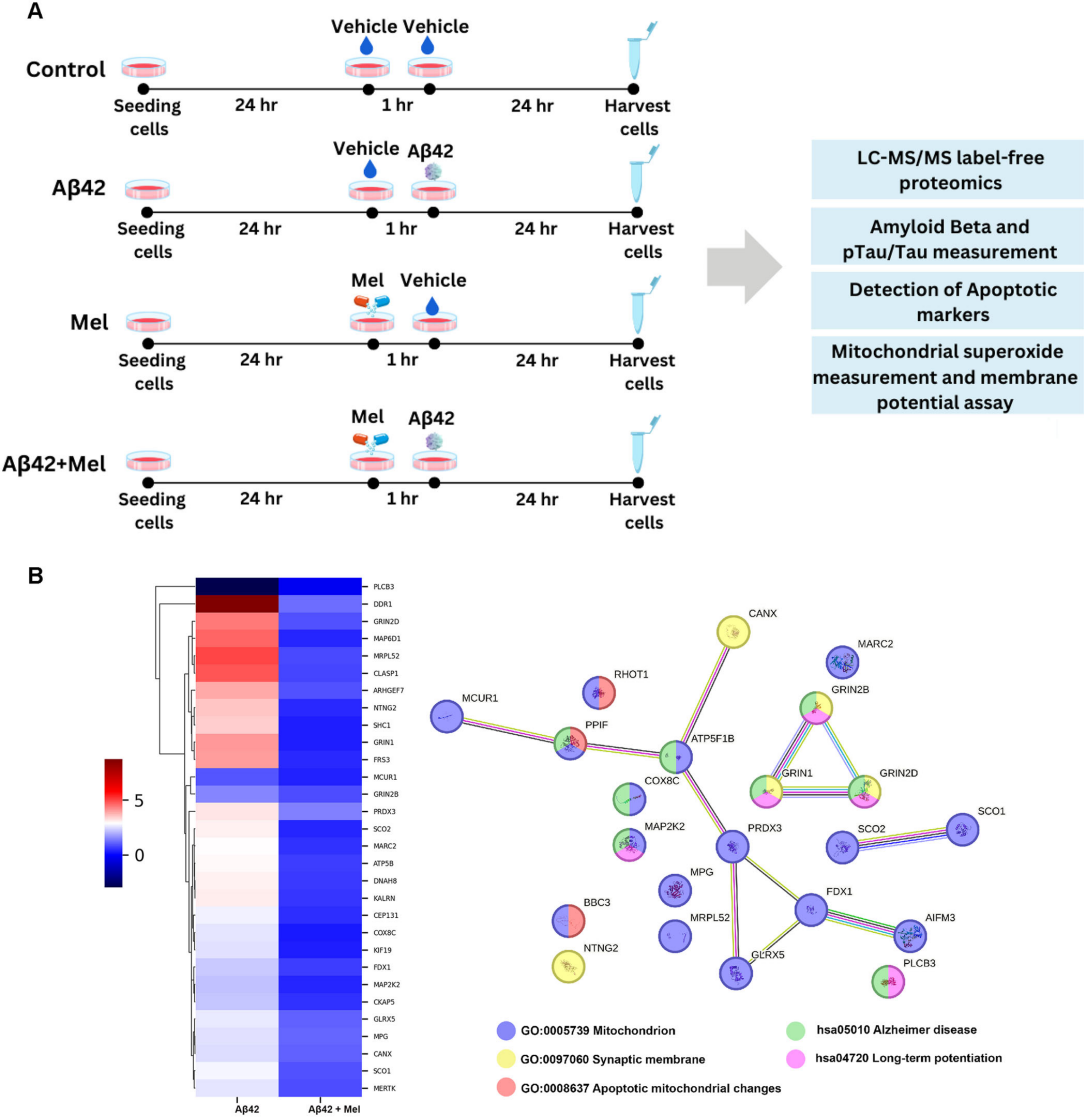
Experimental design and the hierarchical clustering heat map of biologically relevant proteins of SH‐SY5Y cells exposed with Aβ42. (A) Experimental design showing four treatment groups including the control, Aβ42, melatonin, and Aβ42+melatonin. SH‐SY5Y cells were treated with Aβ42 at 1 μM for 24 h with or without 1 h Pretreatment with 1 μM melatonin. Cells were then harvested for subsequent experiments (B) Hierarchical clustering heat map illustrating the abundances of 30 proteins in Aβ42‐ and Aβ42+Mel‐treated cells using label‐free nano‐LC‐MS/MS proteomics analysis. Protein expression levels were normalized to the untreated control, and differential expression was clustered based on log2 fold change over control (*n* = 4). Red represents upregulation, and blue represents downregulation of proteins. The proteins related to different pathways were identified using GO term analysis, STRING interaction network, and KEGG databases.

Aβ42 stock solution was prepared following the manufacturer's protocol to obtain a final concentration of 1 mM, which was stored at −80°C until used. To conduct experiments, Aβ42 was diluted in serum‐free medium to a final concentration of 100 µM, and the aggregation form of Aβ42 was induced by incubating the peptide at 37°C for 5 days, as previously described (Chinchalongporn et al. 2018). Human neuroblastoma SH‐SY5Y cells were cultured in MEM and F‐12 (1:1) supplemented with 10% FBS and 100 units/mL of penicillin/streptomycin, and incubated at 37°C with 95% humidity and 5% CO_2_ until they reached 80–90% confluency. The cells were then seeded into 60 mm petri dishes and, after 24 h of growth, they were pretreated with freshly prepared melatonin at a final concentration of 1 μM. Following a 1‐h incubation period, the cells were treated with Aβ42 at a final concentration of 1 µM for 24 h. The cell pellets were then washed three times with PBS and collected for subsequent experiments, as shown in Figure 1A.

### 2.3 Human Beta‐Amyloid and Tau Protein Measurement

Human Aβ40, Aβ42, total Tau proteins, and phosphorylated Tau Thr181 (pTau181) were prepared using the 4‐plex kit custom MILLIPLEX Human Amyloid Beta and Tau Magnetic Bead Panel (#HNABTMAG‐68K) according to the manufacturer's protocol. The fluorescent signals were detected using the MAGPIX Luminex's xMAP instrument. Concentrations of each marker were analyzed using MILLIPLEX analyst and Belysa software, relative to the standard curve.

### 2.4 Western blot Analysis

Cells were collected and lysed in cold RIPA lysis buffer supplemented with 1% protease and phosphatase inhibitor after Aβ42 treatment (Figure 1A). The total protein concentration for each sample was then measured using Bradford's assay (Bradford 1976). Proteins were separated by SDS‐PAGE using 8–17.5% SDS‐polyacrylamide gels at 120 volts for 1.5 h, as previously described (Panmanee et al. 2015). The membrane was incubated with primary antibodies: mouse anti‐cleaved Caspase 3 (1:1000), rabbit anti‐cleaved PARP (Asp214) (1:1000), rabbit anti‐pGluN2A (1:1000), rabbit anti‐pGluN2B (1:1000), rabbit anti‐GluN2A (1:1000), rabbit anti‐GluN2B (1:1000), and rabbit anti‐vinculin (1:20000) for 1 h at room temperature, and then at 4°C overnight. The membranes were incubated for 5 min with Clarity^TM^ Western ECL substrate. The signals of specific proteins were detected using the Vilber FUSION FX chemiluminescence imaging system.

### 2.5 Label‐Free Proteomics Analysis

Label‐free proteomics analysis was performed and analyzed according to the previously described protocol (Promthep et al. 2022). For protein identification in the discovery phase, raw MS data were analyzed with MaxQuant software (version 1.6.2.10) and its built‐in search engine Andromeda. With the human proteome database retrieved from the UniProt, the default option was set to 1 as a label‐free approach. The label‐free quantitation (LFQ) value minimum ratio count for label‐free quantitation was 1. The false discovery rate (FDR) was fixed at 1% at protein level. With label‐free quantification, the TOF MS/MS match tolerance was set at 0.5 Da. The software's match between run option was utilized to recalibrate mass and retention times between runs. A total of 2,306 unique proteins were identified before the examination of differential expression between control and treatment groups.

### 2.6 ROS Production Assay

Mitochondrial ROS assay was performed using the oxidation‐sensitive fluorescent dye MitoSOX^TM^ Red (Invitrogen). Briefly, SH‐SY5Y cells were seeded in a PhenoPlate™ 96‐well microplate (PerkinElmer) at 3×10^4^ cells per well and incubated for 24 h at 37°C in 5% CO_2_. On the following day, cells were incubated with 5 µM MitoSOX^TM^ Red in serum‐free medium at 37°C for 30 min to detect ROS production. The cells were then washed with PBS twice, and imaged by the Opera Phenix Plus high‐content screening system (PerkinElmer) with the excitation wavelength at 561 nm, an emission wavelength range of 570–630 nm, and a 40× objective capturing 25 images per well. Images were analyzed by Harmony software (PerkinElmer). The change in superoxide production was quantified by the difference in MitoSOX^TM^ Red fluorescence intensity among controls and treated samples. An increase in the fluorescent signal corresponds to an elevation in mitochondrial superoxide levels, indicating mitochondrial dysfunction and cellular damage.

### 2.7 JC‐1 Mitochondrial Membrane Potential Assay

For JC‐1 assay, cells were initially seeded in 96‐well plate at a density of 4×10^4^ cells per well. After 24 h and 90**%** confluence was reached, the cells were treated with or without 1 µM melatonin for 1 h, then with 1 µM Aβ42 for 24 h. Then, 2 µM JC‐1 dye was added to each well and the cells were incubated at 37°C and 5% CO_2_ for 10–15 min. Cells were washed once with PBS. The fluorescent signal was recorded at 475/590 nm for red fluorescence and 475/530 nm for green fluorescence using a fluorescence plate reader. The means of ratios of red to green fluorescent intensities were determined.

For JC‐1 fluorescent staining, after 24 h of treatment, cells were washed once with PBS and then 2 µM JC‐1 dissolved in culture medium was added to each well. After incubation for 20 min, cells were fixed by 4% paraformaldehyde in 0.1 M PBS for 20 min at room temperature and washed with PBS. The staining was then visualized under a confocal laser scanning microscope (FV3000, Olympus, Tokyo, Japan).

### 2.8 Apoptotic Assay

Following the manufacturer's instructions, the Muse^TM^ Annexin‐V & Dead Cell Assay Kit was used to quantitatively analyse the apoptotic profile of Aβ42‐treated SH‐SY5Y cells in the presence or absence of melatonin Pretreatment. Initially, 2.5 × 10^5^ cells/well of SH‐SY5Y cells were seeded into 6‐well plates. After 24 h, the treatment was performed as illustrated in Figure 1A. Following harvesting, cells were centrifuged at 1000 rpm for 5 min, washed in ice‐cold PBS, and then resuspended in serum‐free medium. Following staining with 100 µL of Muse^TM^ Annexin‐V & Dead Cell Reagent, the cell suspensions were left to incubate for 20 min at room temperature in the dark. The Muse^TM^ Cell Analyzer was then used to examine the events for live, early apoptotic, late apoptotic, and dead (necrotic cells).

### 2.9 Statistical and Pathway Analysis

Statistical analysis was conducted using one‐way analysis of variance (one‐way ANOVA) and Tukey's post hoc test with GraphPad Prism version 9 (Graph‐Pad Software Inc., USA). Results with a *p*‐value less than 0.05 were considered statistically significant. Data were expressed as a mean with a standard error of the mean (SEM), and all experiments were performed in at least in triplicates.

For proteomics analysis, a *p*‐value of the individual proteins was calculated and corrected for multiple testing with the Bonferroni correction. By comparing with the control, the proteins that were differentially expressed in the Aβ42‐treated group but not significantly different in the Aβ42+Mel‐treated group were selected to perform the functional analysis. The specific proteins associated with Alzheimer's disease, neuronal synapse, mitochondria, cellular apoptosis, and microtubule‐associated proteins were classified based on the Gene Ontology (GO) term. The list of proteins of interest was then subjected to protein‐protein interaction and pathway analysis using STRING database (www.string-db.org).

## 3 Results

### 3.1 Effect of Melatonin on Proteins Involved in Neuronal Synapses and Alzheimer's Disease‐Related Pathways

**Figure 2:**
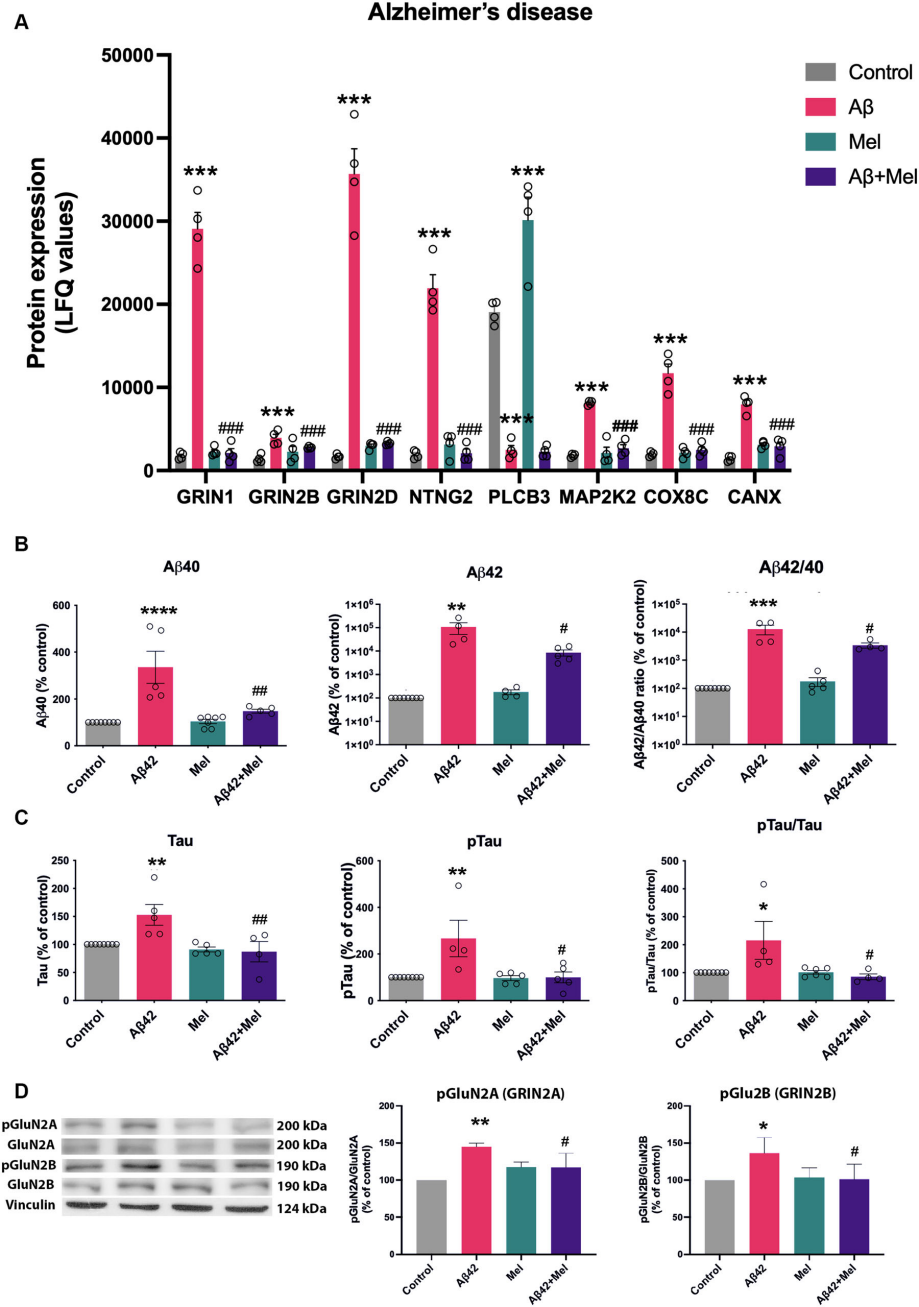
Effect of melatonin and Aβ42 treatments on expression levels of proteins involved in Alzheimer's disease‐related pathways and neuronal synapse. SH‐SY5Y cells were treated with Aβ42 at 1 μM for 24 h with or without Pretreatment with 1 μM melatonin. (A) Label‐free proteomics analysis was used to identify protein expression levels related to synaptic proteins and Alzheimer's pathways, as shown by LFQ quantification. (B–C) The MILLIPLEX MAP Human Amyloid Beta and Tau Panel was used for simultaneous quantification of Aβ40, Aβ42, total Tau (Tau), and phosphorylated Tau Thr181 (pTau181) proteins. (B) Displays the levels of Aβ40, Aβ42, and Aβ42/40 ratio. (C) Shows the levels of total Tau, pTau181, and pTau181/Tau. (D) The expression levels of phosphorylated GluN2A (pGluN2A) and pGluN2B protein expression were measured by Western blot. The band densities were normalized to vinculin and the total GluN2A and GluN2B expression, respectively. The ratios were calculated as a percentage of the control. Data represent the mean ± SEM (*n* = 4–7). *, **, ***, and **** denote significant differences at *p* < 0.05, *p* < 0.01, *p* < 0.001, and *p* < 0.0001, respectively compared to the untreated control group. ^#^, ^##^, and ^###^ denote significant differences at *p* < 0.05, *p* < 0.01, and *p* < 0.001, compared to the Aβ42‐treated group alone.

To evaluate the effect of melatonin on Aβ42‐exposed SH‐SY5Y cells, the altered protein expression of Aβ42‐treated cells in the presence or absence of melatonin Pretreatment was carried out using label‐free nano‐LC‐MS/MS proteomics. Thirty differentially expressed proteins (DEPs) related to the Alzheimer's disease pathway, neuronal synapses, mitochondrial organization, cellular apoptosis, and microtubule‐associated proteins were identified based on GO terms (Figure 1B). The proteins related to the Alzheimer's disease pathway (KEGG: hsa05010) and neuronal synapses (GO: 0097060) were identified using STRING interaction network and KEGG databases (Figure 1B). Among these proteins, three subtypes of N‐methyl‐d‐aspartate (NMDA) receptors including GRIN1 (GluN1), GRIN2B (GluN2B) and GRIN2D (GluN2D) showed significant increases from exogenous Aβ42 treatment (Figure 2A). These three proteins, identified in the Alzheimer's disease pathway, are involved in neuronal synapse formation and long‐term potentiation (Figure 1B). Levels of phosphorylated Tyr1246 GluN2A and Tyr1472 GluN2B, which potentiate NMDA receptor currents and membrane stabilization, were also increased by exposure to Aβ42 (Figure 2D). Pretreatment with melatonin before Aβ42 exposure resulted in the downregulation of NMDA receptor subunits GRIN1, GRIN2B, and GRIN2D, as well as phosphorylated GluN2A and GluN2B, compared to the treatment with Aβ42 alone (Figures 2A and 2D). Furthermore, the administration of exogenous Aβ42 significantly increased the expression of NTNG2, a protein involved in axon guidance and synapse formation, while decreasing the expression of PLCB3, a protein involved in synaptic plasticity processes (Figure 2A). Markedly, Pretreatment with melatonin resulted in a significant decrease in NTNG2 levels (Figure 2A). However, melatonin treatment did not restore the protein expression levels of PLCB3 (Figure 2A). Additionally, a MEK/ERK signaling molecule, MAP2K2, along with COX8C, and an endoplasmic reticulum molecular chaperone, CANX, were significantly increased in Aβ42‐treated group (Figure 2A). Significantly, Pretreatment with melatonin could downregulate these protein expressions to the control levels.

Our results also showed that exogenous Aβ42 significantly enhanced the accumulation of intracellular Aβ40, Aβ42, and Aβ42/40 ratios (Figure 2B). Intracellular levels of Aβ40 increased by 335.5 ± 68.2% (*p* < 0.001), while Aβ42 level increased by three orders of magnitude (*p* < 0.01). In addition, Aβ42 exposure led to a significant increase in total Tau, pTau181, and the pTau181/Tau ratio (Figure 2C). Tau and pTau181 levels increased to 152.9 ± 18.6% (*p* < 0.01) and 266.1 ± 78.3% (*p* < 0.01) respectively, upon exposure to Aβ42. Melatonin Pretreatment significantly reduced the elevated levels of Aβ40 (*p* < 0.01), Aβ42 (*p* < 0.05), and Aβ42/40 (*p* < 0.05), compared to the group treated with Aβ42 alone. Interestingly, melatonin Pretreatment attenuated the increases in Tau (*p* < 0.01), pTau181 (*p* < 0.05), and pTau181/Tau (*p* < 0.05) levels to those observed in the control group.

### 3.2 Neuroprotective Effects of Melatonin Against Aβ42‐induced Neuronal Death via Mitochondria‐Mediated Apoptosis

**Figure 3:**
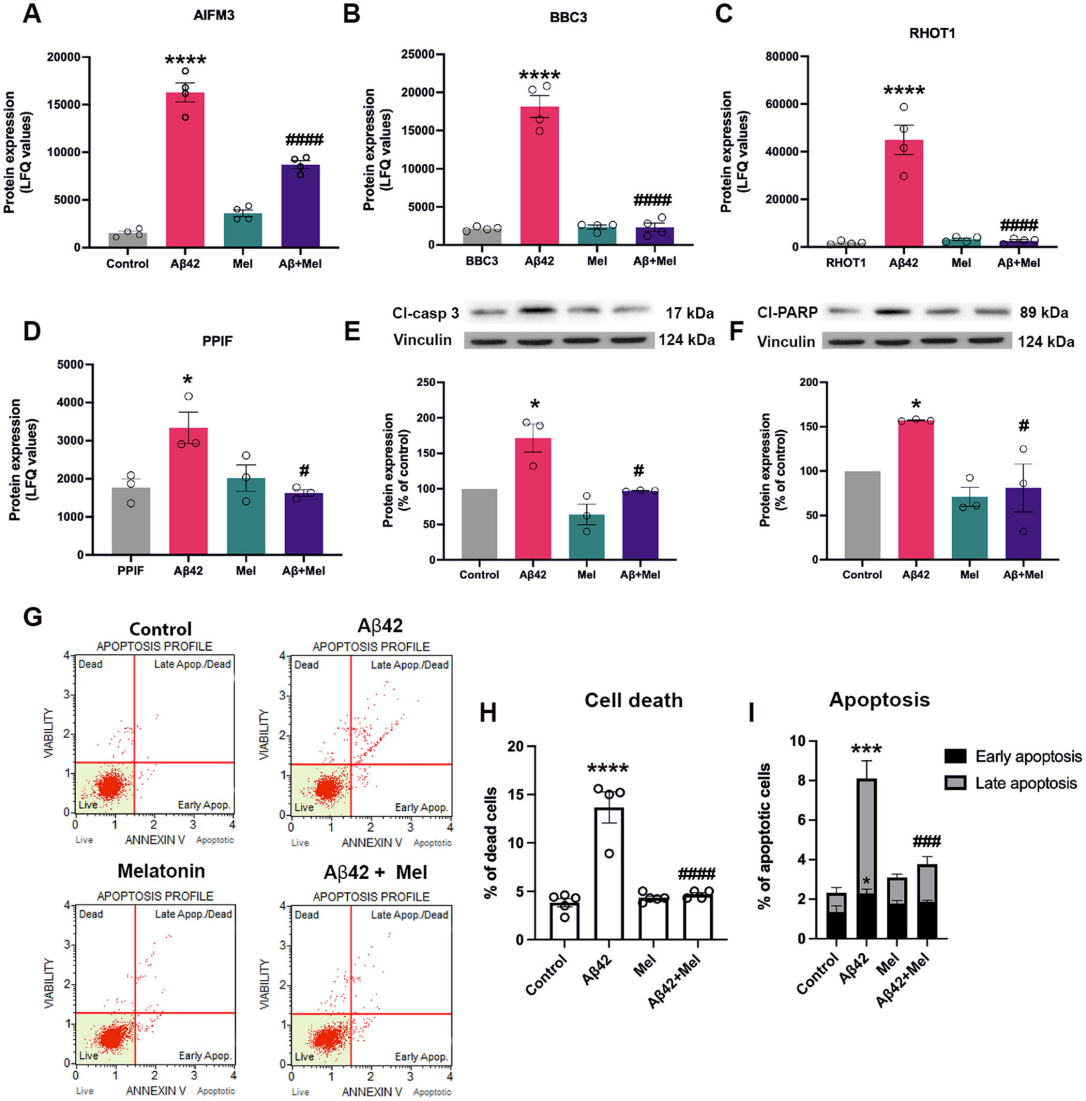
Effect of melatonin on Aβ42‐induced neuronal death via mitochondria‐mediated apoptosis. (A–D) Protein expression levels related to apoptosis pathways were quantified using LFQ quantification. (E–F) To determine the expression levels of Cl‐caspase 3 and Cl‐PARP, Western blot analysis was performed. The band densities were normalized to vinculin and the ratios were calculated as a percentage of the control group's respective value. (G–I) Detection of apoptosis was carried out using Muse^TM^ annexin‐V & dead cell assay kit on SH‐SY5Y cells treated with Aβ42 at 1 μM for 24 h with or without 1 h Pretreatment with 1 μM melatonin. Data are presented as means ± S.E.M (n = 3–4). *, ***, and **** denote statistical significance at *p* < 0.05, *p* < 0.001 and *p* < 0.0001, respectively, compared to the control group. ^#, ###, ####^ shows statistical difference at *p* < 0.05, *p* < 0.001, and *p* < 0.0001, compared to the Aβ42‐treated group alone.

As shown in Figure 1B, Aβ42 treatment upregulated proteins involved in mitochondrion (GO:0005739) and apoptotic mitochondrial changes (GO:0008637) including AIFM3, BBC3, RHOT1, and PPIF. Pretreatment with melatonin significantly reduced the levels of these protein expressions (Figure 3A–D). Mitochondria‐mediated caspase activation in the exogenous Aβ42‐treated cells with or without melatonin Pretreatment was confirmed by Western blot analysis. We found that caspase 3 protease, an apoptotic executioner caspase, was activated in Aβ42‐treated cells, resulting in the generation of an active, cleaved form of caspase 3 (Cl‐caspase 3) (Figure 3E). Furthermore, Cl‐PARP, one of the apoptosis markers and the primary substrate of Cl‐caspase 3, was also found to be upregulated in Aβ42‐treated cells (Figure 3F). In addition, Pretreatment with melatonin significantly reduced the levels of Cl‐caspase 3, Cl‐PARP, as well as Aβ42‐induced cell death and apoptosis (Figure 3G‐I) compared to Aβ42 treatment alone.

### 3.3 Proteomics Analysis Reveals Effects of Melatonin on Aβ42‐induced Changes in Proteins Related to Mitochondrial Organization and ROS Generation

**Figure 4:**
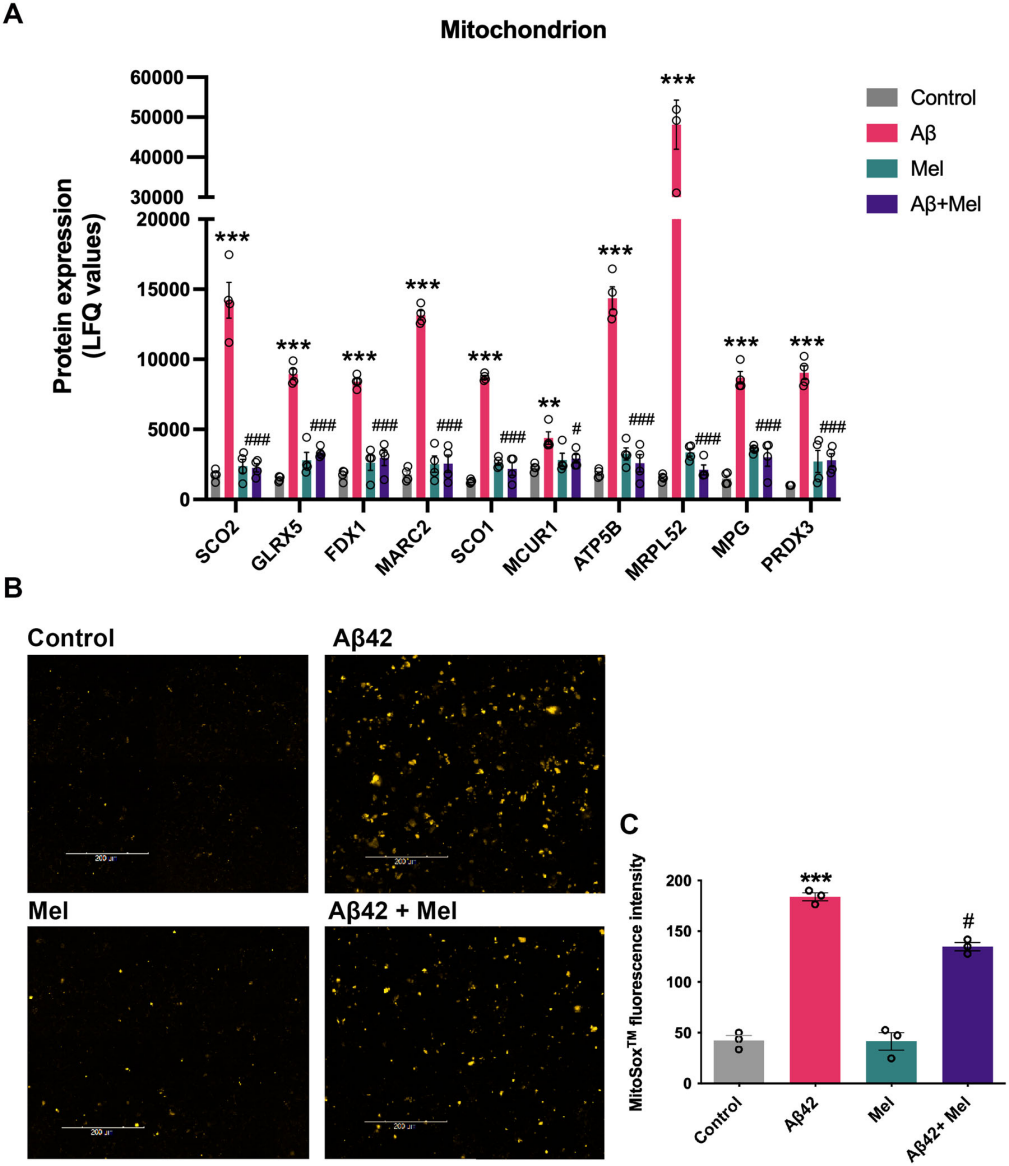
Effect of melatonin on Aβ42‐induced changes in proteins related to functional mitochondrial organization and mitochondrial oxidative stress. SH‐SY5Y cells were treated with Aβ42 at 1 μM for 24 h with or without Pretreatment with 1 μM melatonin. (A) The levels of protein expression related to mitochondrial organization are shown by LFQ values. Data represent the mean ± SEM (*n* = 4). ** and *** denote significant differences at *p* < 0.01 and *p* < 0.001, compared to the untreated control. # and ### denote significant differences at *p* < 0.05 and *p* < 0.001, compared to the Aβ42‐treated group alone. (B) Mitochondrial superoxide levels were measured using the MitoSOX^TM^ assay kit. Cells were imaged by the Opera Phenix Plus high‐content screening system (PerkinElmer). The fluorescent images of the control, Aβ42, Mel and Aβ42+Mel‐treated cells, respectively, are demonstrated. (C) Quantitative measurements of the MitoSOX^TM^ intensity of the control, Aβ42, Mel and Aβ42+Mel treated cells are shown. Data represent the mean ± SEM (*n* = 3). *** denotes significant differences at *p* < 0.001, compared to the untreated control. # denotes significant differences at *p* < 0.05, compared to the Aβ42‐treated group alone.

In our proteomics study, we observed alterations in several proteins involved in functional mitochondrial organization (GO:0005739). Mitochondrial proteins that regulate the opening of the mitochondrial permeability transition pore (mPTP), including BBC3 and PPIF, (Figures 3B and 3D) were upregulated in cells exposed to Aβ42. Electron transport chain (ETC)‐related proteins, including SCO2, GLRX5, and FDX1, were increased in cells treated with Aβ42 (Figure 4A). Pretreatment with melatonin was observed to significantly reduce the expression of these proteins to the control levels (Figure 4A).

Other proteins associated with the mitochondrial subcellular compartment, including MARC2, SCO1, MCUR1, ATP5B, and MRPL52, were significantly upregulated in cells treated with Aβ42 (Figure 4A). Pretreatment with melatonin before Aβ42 exposure resulted in the downregulation of these proteins compared to Aβ42 treatment alone (Figure 4A). In addition, Aβ42 exposure resulted in the upregulation of proteins associated with the cellular response to DNA damage stimuli, including MPG and PRDX3, both of which are antioxidant enzymes primarily located within mitochondria (Figure 4A). Interestingly, Pretreatment with melatonin downregulated both MPG and PRDX3 protein expressions (Figure 4A).

We next evaluated the effect of melatonin on the level of mitochondrial ROS generation under Aβ42 exposure. SH‐SY5Y cells were treated with or without 1 µM melatonin before incubation with 1 µM Aβ42. Then, the MitoSOX^TM^ assay was employed to assess mitochondrial superoxide levels in the viable cells. A fluorescent dye, MitoSOX^TM^, is selectively localized in mitochondria. Upon entering the mitochondria, the dye undergoes oxidation by superoxide, yielding a fluorescent signal. The findings from high‐content imaging analysis revealed a robust fluorescent signal in cells treated with Aβ42, indicating a significant increase in superoxide species levels (Figure 4B). In contrast, Pretreatment with 1 µM melatonin resulted in a less pronounced level of superoxide staining (Figure 4B). The control group and cells treated with melatonin alone exhibited minimal superoxide staining across all fields (Figure 4B). Additionally, quantitative analysis of MitoSOX^TM^ fluorescence intensity demonstrated a significant increase in mitochondrial superoxide levels in cells treated with 1 µM Aβ42, with a mean value of 183.96 ± 6.80 arbitrary units (AU) (*p* < 0.001) compared to the control group (Figure 4C). However, Pretreatment with 1 µM melatonin resulted in a significant reduction in MitoSOX^TM^ intensity at 134.88 ± 7.07 AU (*p* < 0.05), compared to Aβ42 treatment alone (Figure 4C).

### 3.4 Melatonin‐Mediated Neuroprotection Against Aβ42‐induced Membrane Depolarization

**Figure 5:**
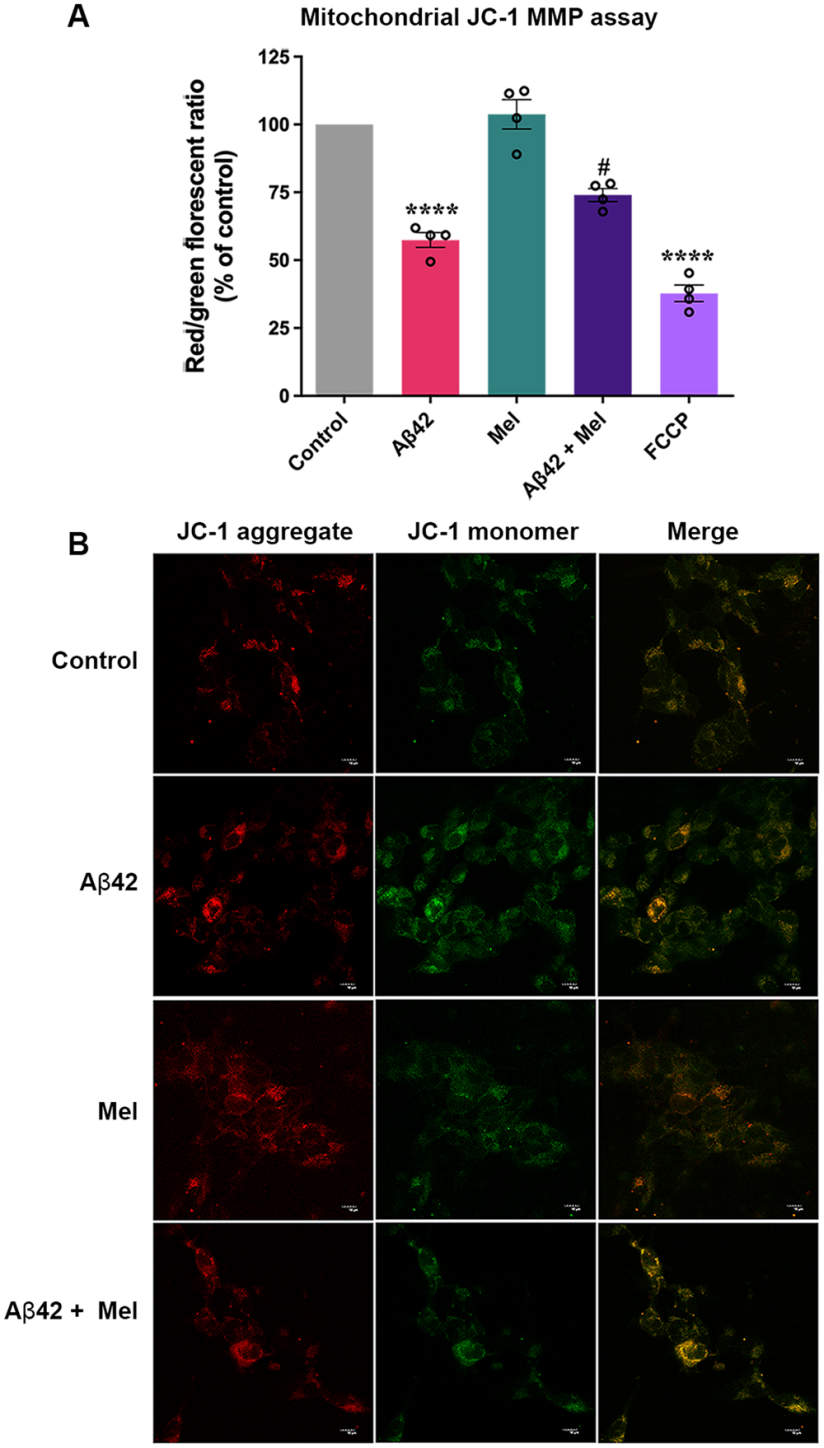
Effect of melatonin on Aβ42‐induced mitochondrial membrane potential (MMP) impairment. (A) The JC‐1 membrane potential assay was used as a standard marker for an intact mitochondrial membrane potential. The red to green fluorescence ratios represent aggregate/monomer JC‐1 levels, while a higher fluorescent ratio indicates intact MMP. Data represent the mean ± SEM (*n* = 4). **** denotes significant differences at *p* < 0.0001, compared to the untreated control. # denotes a significant difference at *p* < 0.05, compared to the Aβ42‐treated group alone. (B) Immunofluorescence staining with JC‐1 was employed to visualize and compare MMP in control, Aβ42, Mel, and Aβ42+Mel treated cells.

To further evaluate melatonin's protective ability on Aβ42‐induced mitochondrial activity impairment, we assessed the mitochondrial membrane potential (MMP) using the JC‐1 assay. The results demonstrated that the JC‐1 ratio, determined by the ratio of JC‐1 aggregates (red) to JC‐1 monomer (green), significantly decreased to 57.43 ± 5.48% (*p* < 0.0001) in Aβ42‐treated cells compared to the control group (Figure 5A). This indicates an increase in mitochondrial membrane depolarization. In contrast, Pretreatment with melatonin resulted in significantly more JC‐1 aggregates compared to Aβ42 treatment alone, at a level of 73.99 ± 4.77% (*p* < 0.05) (Figure 5A). Additionally, the administration of FCCP, a potent uncoupler of mitochondrial oxidative phosphorylation serving as a positive control for mitochondrial membrane depolarization, significantly decreased aggregated JC‐1 levels to 37.79 ± 6.05% (*p* < 0.0001) compared to the control group (Figure 5A). To further confirm the effect of melatonin against Aβ42‐induced MMP impairment, an immunofluorescence staining was conducted, as shown in Figure 5B. Overall, our results indicate that melatonin may exert a protective effect against Aβ42‐induced mitochondrial impairment by mitigating mitochondrial ROS generation and preventing membrane depolarization, thus leading to the preservation of mitochondrial function.

### 3.5 Effect of Melatonin on Exogenous Aβ42‐induced Changes in Microtubule Biological Processes, Tyrosine Kinase Receptor Signaling, and Rho GTPase Signaling

**Figure 6:**
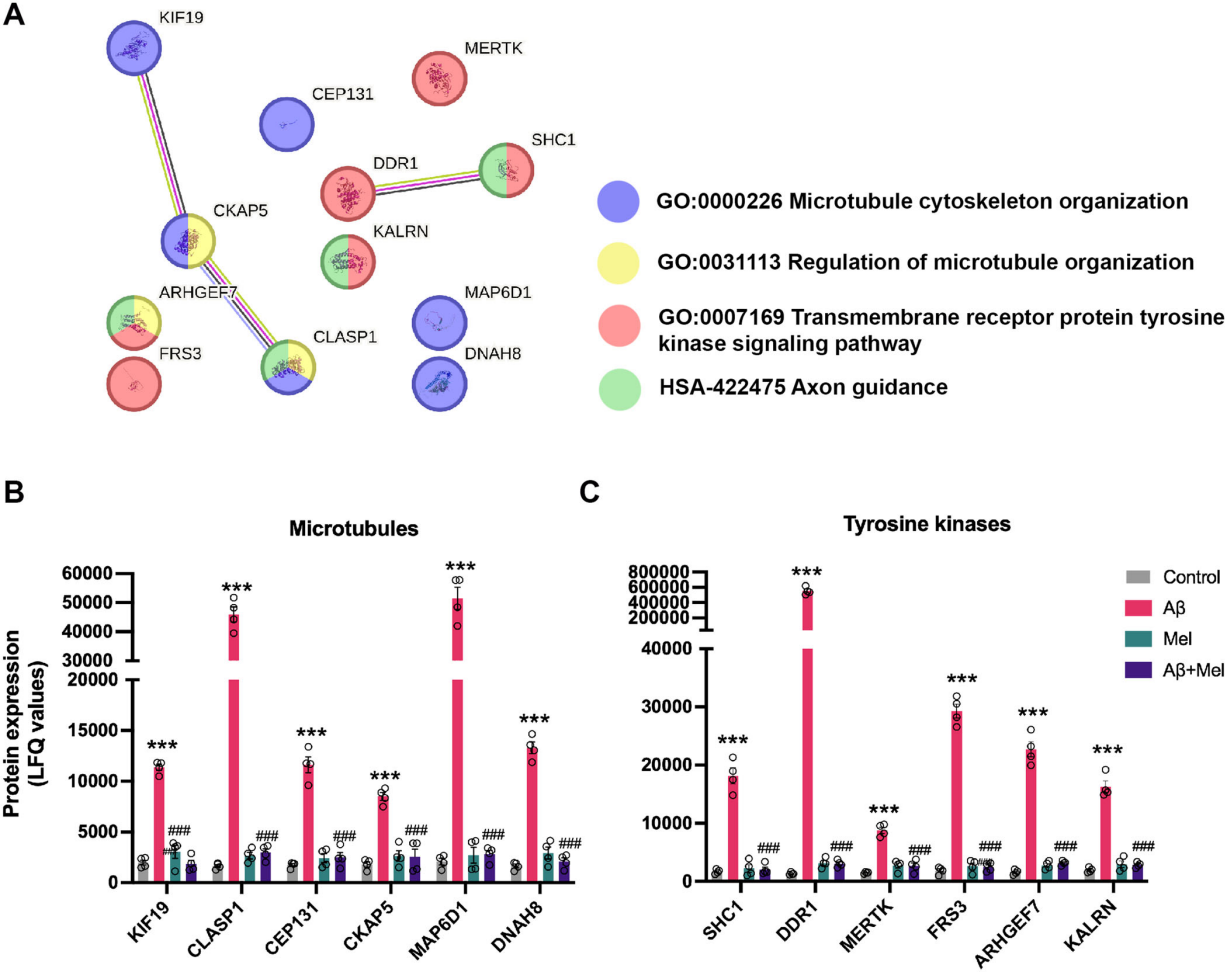
Effect of melatonin on exogenous Aβ42‐induced changes in microtubule biological processes and signal transductions involving tyrosine kinase signaling. SH‐SY5Y cells were treated with Aβ42 at 1 μM for 24 h with or without Pretreatment of 1 μM melatonin. (A) The STRING interaction network of proteins involved in microtubule biological processes and SHC1 related tyrosine kinase signaling were identified using GO terms and protein‐protein interactions. The levels of protein expression related to (B) microtubule biological processes, (C) tyrosine kinase signaling, and Rho GTPase signaling were quantified using LFQ quantification. Data represent the mean ± SEM (*n* = 4). * and *** denote significant differences at *p* < 0.05 and *p* < 0.001, compared to the untreated control. ### denotes significant differences at *p* < 0.001, compared to the Aβ42 treatment alone.

We have identified changes in several microtubule‐related proteins that are involved in microtubule organization and stabilization in Aβ42‐treated cells (Figure 6A). The identified proteins responsible for microtubule organization, comprised of KIF19, CLASP1, CEP131, CKAP5, MAP6D1, and DNAH8, were significantly upregulated in Aβ42‐treated cells (Figure 6B). Upon Pretreatment with melatonin, the levels of these proteins were significantly decreased to the same level as the control group (Figure 6B).

We further investigated the tyrosine kinase receptor clusters associated with hyperphosphorylated tau NFTs. The first group of proteins, including SHC1, DDR1, MERTK, and FRS3, were upregulated upon Aβ42 exposure, and melatonin was shown to significantly impede this upregulation (Figure 6C). For regulation of microtubule organization, various GTPases are also involved in the pathways, especially Rho GTPase signaling. We investigated the expression levels of proteins associated with this pathway. ARHGEF7 and KALRN, a protein assisting in the exchange of GDP by GTP, were upregulated in cells exposed to Aβ42 (Figure 6C), where Pretreatment with melatonin resulted in a significant decrease in the expression levels of these proteins (Figure 6C).

## 4 Discussion

Neuropathological hallmarks of Alzheimer's disease, including extracellular depositions of β‐amyloid plaques and intracellular accumulation of NFTs composing tau proteins, have been well characterized (Castellani et al. 2010). These abnormal protein accumulations lead to synaptic loss and eventually neuronal cell death (Serrano‐Pozo et al. 2011). Melatonin concentration ranging from 10 nM to 10 μM has been reported to inhibit Aβ‐induced neuronal apoptosis and mitochondrial dysfunction (Cheng et al. 2006), while higher pharmacological concentrations (μM–mM ranges) have been implicated in antioxidant and free radical scavenger effects (Martín et al. 2002). In contrast, at pM–nM concentration, melatonin could bind and activate its G protein‐coupled membrane receptors and downregulate the expression levels of β‐ and γ‐secretases, resulting in the inhibition of Aβ generation (Panmanee et al. 2015; Tarocco et al. 2019). Using LC‐MS/MS label‐free proteomics, we found that Pretreatment with 1 μM melatonin could protect SH‐SY5Y cells against Aβ42‐induced cellular damages by several mechanisms, including regulating the expression of proteins involved in the AD pathway, neuronal synapses, mitochondrial functions and microtubule biological processes (Figure 1B).

We identified several proteins involved in the Alzheimer's disease pathway, including NMDA receptor (NMDARs) subunits (GRIN1, GRIN2B, and GRIN2D) (Figure 2A). The brain regions predominantly affected by AD exhibited NMDARs primarily composed of GRIN2A (GluN2A) and GRIN2B (GluN2B), while extrasynaptic NMDARs, containing GRIN2B subunits, have been linked to neuronal excitotoxicity (Hardingham and Bading 2010). The induction of excitotoxicity and synaptic dysfunction caused by excessive Aβ oligomers was dependent on the glutamatergic NMDA receptors (De Felice et al. 2007). Consistently, we found an upregulation of GRIN2B, GRIN1, and GRIN2D, in response to exogenous Aβ42. In cultured neurons, Aβ oligomers elevated GluN2B levels in neuronal membranes and enhanced NMDA‐induced intracellular Ca^2+^ levels in an integrin β1 and PKC‐dependent pathway (Kodis et al. 2018; Ortiz‐Sanz et al. 2022). Moreover, we observed that Aβ42 exposure increased levels of pGluN2A (Tyr1246) and pGluN2B (Tyr1472), indicating NMDA receptor activation. Phosphorylation at Tyr1472 GluN2B is associated with NMDA receptor‐induced Ca^2+^ influx in neuronal ischemic and NMDA‐induced excitotoxicity models (Knox et al. 2014; Li et al. 2017). Our study showed that Pretreatment with melatonin significantly reduced the overexpression of these glutamate receptor subtypes and the phosphorylated forms induced by Aβ42 exposure (Figures 2A and 2D). Consistently, melatonin has been found to inhibit NMDA‐mediated neuronal excitation by regulating neuronal nitric oxide synthase and mPTP (Escames et al. 2004; Furuta et al. 2022). Excessive activation of these receptors may ultimately lead to excitotoxicity and synaptic dysfunction. Aβ‐induced excessive influx of calcium in postsynaptic neurons has been reported to trigger excitotoxicity, which in turn initiates a series of events including increased ROS production, tau phosphorylation, and mitochondrial dysfunction, ultimately leading to synaptic dysfunction (Ferreira et al. 2015). Additionally, we found that the levels of MAP2K2 (MEK2) and COX8C were upregulated upon Aβ42 exposure, and melatonin treatment could decrease the expression levels of these proteins to the control levels (Figure 2A). Inhibiting MEK was reported to protect neurons from the accumulation of Aβ by promoting autophagic lysosomal activity (Chun et al. 2022). Exposure to Aβ42 led to an increase in CANX, an ER molecular chaperone, reported to co‐localize with tau and pTau in AD brains (Meier et al. 2015). Interestingly, Pretreatment with melatonin was observed to reduce the level of CANX to control levels (Figure 2A).

Intraneuronal Aβ accumulation has also been reported in AD, which involves the development of synaptic pathology and senile plaques, which in turn results in elevated tau hyperphosphorylation and mitochondrial dysfunction (Pavlov et al. 2009). We found that Aβ42 exposure significantly increased intraneuronal ratios of Aβ42/40 and pTau181/total Tau, which are the hallmarks of AD pathology (Wang et al. 2021). Pretreatment with melatonin significantly prevented these accumulations and their corresponding ratios (Figure 2B–C). The previous study showed that melatonin administration suppressed tau phosphorylation in mice hippocampi via the PI3K/Akt/GSK3β signaling pathway (Ali and Kim 2015). The enhanced accumulations of Aβ and phosphorylated tau have been reported to interfere with microtubule stability and had a detrimental effect on axonal trafficking and dendritic functions (Barredo and Balanay 2023; Pianu et al. 2014). In addition, melatonin was previously shown to inhibit Aβ production via inhibition of β‐ and γ‐secretase enzymatic activities in SH‐SY5Y cells (Chinchalongporn et al. 2018; Panmanee et al. 2015).

We also investigated the neuroprotective effects of melatonin on Aβ42‐induced neuronal death via mitochondria‐mediated apoptosis. Upon exposure to various forms of cellular stress, such as oxidative stress and protein misfolding, the apoptosis‐inducing factor AIFM3 and the p53 upregulated modulator of apoptosis (PUMA or BBC3) could become activated and induce neuronal apoptosis (Galehdar et al. 2010). AIFM3 has been found to co‐localize with NFTs and increase in the hippocampus and entorhinal cortex of AD patients (Yu et al. 2010). Consistently, we observed the upregulation of AIFM3 and BBC3 in Aβ42‐exposed cells, and Pretreatment with melatonin could significantly suppress the levels of these proteins (Figure 3A–B). We also found that melatonin could reverse the enhanced levels of mitochondrial proteins, namely RHOT1 (mitochondrial outer membrane) and PPIF (mPTP regulating mitochondrial inner membrane) in Aβ42‐exposed cells (Figure 3C–D). The depletion of PPIF expression has been reported to protect against Aβ‐induced ROS production and toxicity on the motility and dynamics of axonal mitochondria via the inhibition of the p38 MAPK signaling pathway (Guo et al. 2013). Furthermore, we also evaluated the effect of melatonin treatment on levels of Cl‐caspase 3 and Cl‐PARP in Aβ42‐exposed cells. Upon activation, caspases play a crucial role in triggering cellular apoptosis by cleaving and activating effector caspases that subsequently execute the apoptotic program. Among the various cellular substrates of caspases, PARP is known to be one of the targets of caspases and deemed as a hallmark of apoptotic cell death (Chaitanya et al. 2010). We found that melatonin Pretreatment significantly reduced the levels of Cl‐caspase 3 and Cl‐PARP in Aβ42‐treated cells (Figure 3E–F). Melatonin was shown to suppress apoptotic cell death by inhibiting caspase activation, cytochrome c, and PARP1 via melatonin receptor 1 A (MT1A) (Ali and Kim 2015; Suofu et al. 2017; Zhang et al. 2013). Antiapoptotic properties of melatonin are mediated via NF‐κB inhibition and Akt signaling activation (Yang et al. 2020; Zhi et al. 2020). Moreover, Aβ peptides were also reported to induce the formation of tau filaments in neurons through the activation of caspases that cleave tau at the C‐terminus in Aβ42‐exposed cells (Gamblin et al. 2003). This process generates a proteolytic fragment, which in turn promotes faster polymerization kinetics and exacerbates AD pathology (Abraha et al. 2000).

We also demonstrate the potential of melatonin as a neuroprotective agent against Aβ42‐induced mitochondrial impairment. Recent findings have shown that mitochondria can synthesize melatonin within the mitochondrial matrix (Suofu et al. 2017). Additionally, previous studies have found that Aβ peptides could accumulate within the mitochondrial matrix, leading to disrupted mitochondrial function and oxidative stress (Chen and Yan 2007). Melatonin may protect mitochondria against oxidative stress in AD pathology (Tan et al. 2013, 2023). Our findings showed that melatonin Pretreatment prevented mitochondrial ROS generation and prevented membrane depolarization, leading to the preservation of mitochondrial function (Figures 4, 5). Upon Aβ42 exposure, we found changes in several proteins involved in the mitochondrial ETC, such as SCO2, GLRX5, and FDX1 (Figure 4A). We also found an increase in mitochondrial oxidative stress under Aβ42 exposure using the MitoSOX^TM^ assay, while Pretreatment with melatonin suppressed mitochondrial ROS production (Figure 4B). Furthermore, Aβ42 exposure resulted in mitochondrial membrane depolarization, while Pretreatment with melatonin could decrease the degree of mitochondrial membrane depolarization (Figure 5).

Neuronal microtubules function as a modulator in the development and long‐term maintenance of neurons; and tau protein is central to microtubule growth, assembly, and stabilization. These microtubule functions depend on the dynamic between polymerized and depolymerized forms (Peris et al. 2022). Indeed, neuronal communications require an optimal ratio of dynamic and stable microtubules, and disturbances in this ratio may lead to neurodegenerative diseases, including AD. Therefore, we investigated changes in the expression of proteins associated with microtubule processes under Aβ42 treatment (Figure 6A–B). Upon the Pretreatment, melatonin's effects may prevent the over formation of microtubules by lessening the levels of these proteins, thereby protecting the dynamic nature of microtubules from further disruption. During Aβ42‐induced cellular stress, melatonin might preserve the cells by limiting the transportation of unwanted cargoes through the downregulation of the protein components in kinesin/dynein complexes such as KIF19 and DNAH8 (Figure 6B). Having observed the overexpression of microtubule‐related proteins under Aβ42‐induced cellular stress, we then investigated Rho signaling, as this pathway is reported to be implicated in AD pathologies and microtubule stabilization (Aguilar et al. 2017). There are two main players in regulating Rho GTPase signaling: Rho guanine nucleotide exchange factor (GEF) and Rho GTPase‐activating protein (GAP) (Cook et al. 2014). The regulation of Rho GTPase signaling demonstrates the interplay between GEFs and GAPs, in which GEFs promote Rho GTPase activity, while GAPs inhibit it (Cai et al. 2021). We found that the overexpression of GEFs, including ARHGEF7 and KARLN in Aβ42‐induced cells, could be reversed by melatonin Pretreatment (Figure 6C). Overall, exogenous Aβ42 promoted several proteins involved in microtubule formation, unbalancing the Rho GTPase pathway.

**Figure 7:**
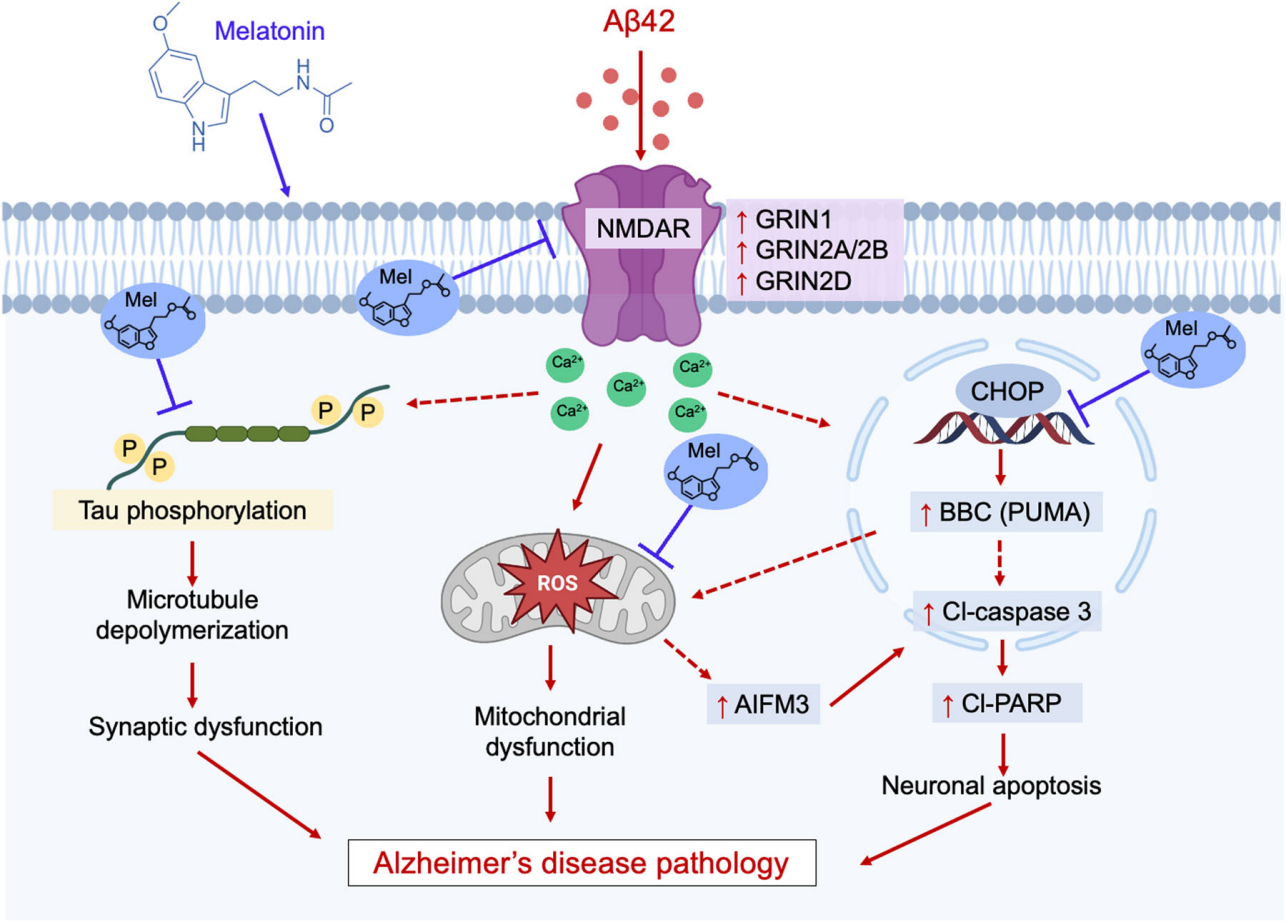
The proposed mechanisms for melatonin's role in preventing AD pathology. Aβ42 exposure induced increases in intracellular accumulation of Aβ42/40, pTau (Thr181)/Tau ratios, mitochondrial ROS production, and mitochondrial membrane depolarization. Several protein markers associated with cellular apoptosis and mitochondrial functions were changed, including permeability transition pore (mPTP) regulators and the electron transport chain (ETC). Melatonin could modulate a variety of cellular processes giving rise to its promising therapeutic potential in the treatment and prevention of AD. Images adapted from Biorender. com.

Taken together, our findings support the hypothesis that the intraneuronal NFTs accumulation, influenced by extracellular Aβ42 and the increase in intraneuronal APP cleavage products, for example, amyloid proteins, can cause neuronal toxicities which leads to the progression of AD pathogenesis (Bi et al. 2019; Lewis et al. 2001). Treatment with Aβ42 led to alterations in protein expression associated with cellular responses to oxidative stress, cellular apoptosis, and mitochondrial dysfunction. These changes include modifications in antioxidant enzymes, proteins regulating the mPTP, and the ETC, as well as proteins within the mitochondrial subcellular compartment. Pretreatment with melatonin was observed to modulate several mitochondrial proteins, suggesting it has potential as a therapeutic target for preventing or treating neurodegenerative disorders associated with Aβ42‐induced mitochondrial impairment. In addition, melatonin showed neuroprotective effects by suppressing the overexpression of proteins involved in microtubule formation. Therefore, this study suggests that melatonin has promising therapeutic potential in preventing AD by modulating the proteins that underpin significant cellular processes. A proposed mechanism by which melatonin could ameliorate Alzheimer's disease pathology is illustrated in Figure 7.

## Author Contributions

**Jiraporn Panmanee:** investigation, validation, formal analysis, conceptualization, methodology, supervision, formal analysis, visualization, writing – original draft, writing – review and editing, funding acquisition. **Matthew Phanchana:** investigation, validation, conceptualization, methodology, supervision, writing – review and editing. **Phorutai Pearngam:** investigation, validation, formal analysis, conceptualization, visualization, writing – original draft, writing – review and editing. **Nopphon Petchyam:** formal analysis, visualization, writing – original draft, writing – review and editing. **Kornkanok Promthep:** investigation, validation. **Ponlawit Wisomka:** investigation, validation, formal analysis. **Suchanoot Kutpruek:** investigation, validation. **Supitcha Pannengpetch:** investigation, validation, formal analysis. **Tanya Prasertporn:** investigation, validation. **Sujira Mukda:** investigation, validation, resources, supervision. **Piyarat Govitrapong:** conceptualization, resources, supervision. **Chutikorn Nopparat:** investigation, validation, formal analysis, conceptualization, methodology, supervision, formal analysis, visualization, writing – original draft, writing – review and editing.

## Conflicts of Interest

The authors declare no conflicts of interest.

## Data Availability

The data that support the findings of this study are available from the corresponding author upon reasonable request. The raw data supporting the conclusions of this article are available upon request from the corresponding author.
